# Cultural values as determinants of psychological resilience in Tunisian women

**DOI:** 10.1192/j.eurpsy.2025.1612

**Published:** 2025-08-26

**Authors:** H. Mhiri, I. Chaari, I. Mannoubi, N. Boussaid, F. Charfeddine, L. Aribi, N. Messedi, J. Aloulou

**Affiliations:** 1 Psychiatric department “B”, Hedi Chaker University Hospital, Sfax, Tunisia

## Abstract

**Introduction:**

Resilience, the ability to adapt and thrive amid adversity, is particularly prominent in Tunisian women, who are recognized for their strength and adaptability despite societal pressures. Their resilience appears closely linked to cultural heritage, indicating a significant connection between resilience and cultural values. This study explores how cultural values shape the psychological resilience of Tunisian women.

**Objectives:**

To examine the influence of cultural values on resilience among Tunisian women.

**Methods:**

A cross-sectional survey was conducted Tunisian women aged 18 and above, from June to August 2024. Sociodemographic data were gathered, while cultural values were assessed using the Individual Cultural Values Scale (CVScale) and the Centrality of Religion Scale (CRS-5). Resilience was measured with the 25-item Connor-Davidson Resilience Scale (CD-RISC 25).

**Results:**

We collected 695 responses in our survey. Participants had a mean age of 36.72 ± 12.23 years, with 90.9% holding university degrees, 56.5% employed, 49.2% married, and 50.6% with children. The mean resilience score was 68.26 ± 14.09, with 26.3% showing low resilience. Average scores for cultural values were “power distance” (9.13 ± 3.46), “uncertainty avoidance” (20.84 ± 2.95), “masculinity” (9.55 ± 3.99), “collectivism” (21.64 ± 5.07), “long-term orientation” (25.89 ± 2.94), and “centrality of religion” (3.95 ± 0.77).

Resilience correlated positively with “uncertainty avoidance” (p < 10⁻³, r = 0.145), “collectivism” (p < 10⁻³, r = 0.208), “long-term orientation” (p < 10⁻³, r = 0.267), and “centrality of religion” (p < 10⁻³, r = 0.186). Conversely, resilience was negatively correlated with “power distance” (p < 10⁻³, r = -0.104), and no significant correlation was observed with masculinity.

**Conclusions:**

The findings reveal that cultural values significantly shape resilience in Tunisian women. Higher resilience was associated with uncertainty avoidance, collectivism, long-term orientation, and religious centrality, while power distance negatively impacted resilience. This emphasizes the need to incorporate cultural dimensions when designing initiatives to support resilience among women in Tunisia.

**Disclosure of Interest:**

None Declared

